# Confirmation of Recurrent Lung Cancer Following Resection Using Liquid Biopsy, a Proof-of-Concept Real-World Study

**DOI:** 10.3390/curroncol31070302

**Published:** 2024-07-17

**Authors:** Julia R. Naso, Stephen Yip, Curtis Hughesman, Barb Melosky, Tanner Dowhy, Melissa K. McConechy, John C. English, Penelope M. A. Brasher, James Choi, Kyle Grant, John Yee, Stephen Lam, Anna McGuire

**Affiliations:** 1 Department of Pathology and Laboratory Medicine, University of British Columbia, Vancouver, BC V5Z 4E6, Canada; julia.naso@vch.ca (J.R.N.);; 2 Department of Pathology and Laboratory Medicine, Vancouver General Hospital, Vancouver, BC V5Z 1M9, Canada; 3 Cancer Genetics and Genomics Laboratory, BC Cancer, Vancouver, BC V5Z 4E6, Canada; 4 Department of Pathology, BC Cancer, Vancouver, BC V5Z 4E6, Canada; 5 Department of Medical Oncology, BC Cancer, Vancouver, BC V5Z 4E6, Canada; 6 Canada’s Michael Smith Genome Sciences Centre, Vancouver, BC V5Z 4S6, Canada; 7 Centre for Clinical Epidemiology and Evaluation, Vancouver Coastal Health Research Institute, Vancouver, BC V5Z 1M9, Canada; 8 Division of Thoracic Surgery, Department of Surgery, Vancouver General Hospital, Vancouver, BC V5Z 1M9, Canada; 9 Division of Respirology, Department of Medicine, BC Cancer, Vancouver, BC V5Z 4E6, Canada; 10 Vancouver Coastal Health Research Institute, Vancouver, BC V5Z 1M9, Canada

**Keywords:** lung cancer, next-generation sequencing, biomarker, targeted therapy

## Abstract

Appropriate management requires timely and accurate confirmation of non-small cell lung cancer (NSCLC) recurrence in patients who have had curative-intent surgical resection. We assessed the association between circulating tumor DNA (ctDNA) identified using amplicon sequencing and evidence of recurrence on CT surveillance. A prospective cohort study of NSCLC patients with early-stage disease undergoing curative-intent resection was conducted. Surveillance was performed post-operatively at pre-defined intervals with both liquid biopsy and chest CT imaging. Amplicon panel next-generation sequencing was performed on DNA and RNA from tumor tissue and on plasma cell-free DNA for tumor-informed ctDNA detection. Resected tumors from 78 NSCLC patients were analyzed. Alterations were detected on the DNA assay for 65 tumors and only on the RNA assay for 4 tumors. Of the 65 patients with alterations detected on the tumor DNA assay, 29 completed post-operative liquid biopsy testing. Four of those 29 patients had evidence of recurrence on imaging, of whom two had biopsy confirmation of recurrence and detectable ctDNA at the 12-month follow-up. Molecular confirmation of NSCLC recurrence can be provided through amplicon sequencing of plasma cell-free DNA in cases with imaging evidence of recurrence. Invasive tissue diagnosis may be avoidable in patients with ctDNA confirmation of recurrence that is suspected based on imaging. Further study of ctDNA assessment technologies in the setting of suspected recurrence is necessary to inform post-operative lung cancer surveillance guidelines.

## 1. Introduction

Lung cancer patients with the highest probability of cure are those who present with early-stage non-small cell lung carcinoma (NSCLC; stages I to IIIA) and are amenable to complete surgical resection [1]. However, the five-year overall survival for stage IB disease is estimated at only ~68% [1], with even lower survival for higher-stage disease. Stage IB-IIIA NSCLC may now be treated with neoadjuvant and adjuvant systemic immune or gene-targeted therapies [2,3,4,5], contingent upon the results of molecular testing. Additional targeted treatments may also be administered upon evidence of disease recurrence following resection, necessitating detection and confirmation of recurrent disease.

Following curative intent surgical resection, post-treatment oncologic surveillance at pre-defined intervals with computed tomography (CT) chest imaging is recommended in clinical practice guidelines [6]. The purpose of post-treatment surveillance is to detect treatable second primaryand locally recurrent disease, thereby extending survival. The risk of recurrent malignancy is highest in the first two years following resection, with CT imaging typically performed every six months during the first two years post-operatively [6].

The clinical challenge with CT surveillance is false positives. For instance, following the completion of immune therapy, intrapulmonary and paratracheal lymphadenopathy is common as part of a normal immune response. Furthermore, non-specific bronchial inflammation with common seasonal viral infections can lead to indeterminate false-positive findings of ground-glass and mixed-density nodules that are concerning for recurrence or early second primary malignancy. With the persistence of these abnormalities, the patient may be subject to diagnostic intervention and their accompanying risks to guide further treatment. Biopsy carries risks for the patient associated with sedation, pneumothorax, and bleeding, and the position of some lesions precludes an attempt at biopsy [7]. The diagnostic yield of a tumor biopsy is further complicated by operator experience, and non-diagnostic biopsies result in treatment delays while scheduling a repeat biopsy.

Liquid biopsy offers an appealing alternative to tumor tissue biopsy in the scenario of indeterminate clinical findings on post-operative surveillance CT chest. The identification of circulating tumor DNA (ctDNA) in plasma may provide strong and timely evidence of recurrence and inform on targetable alterations [8], with a reduced risk of serious complications compared to tissue testing. However, methods of detecting ctDNA vary widely in their sensitivity to detect mutations and amenability to in-house adoption. The simple approach of applying the same amplicon-based panel sequencing assay to FFPE tumor tissue and plasma cfDNA may be more easily implemented. These types of assays may not be optimized for highly sensitive minimal residual disease detection but may still be sensitive and specific enough to serve as a useful diagnostic adjunct to CT imaging for the detection of recurrent NSCLC disease following curative-intent resection. The current study aims to explore the co-occurrence of ctDNA detected post-operatively using an amplicon sequencing approach and the clinical finding of a possible recurrence on routine surveillance CT imaging in a real-world clinical setting.

## 2. Materials and Methods

### 2.1. Study Design and Setting

**Figure 1:**
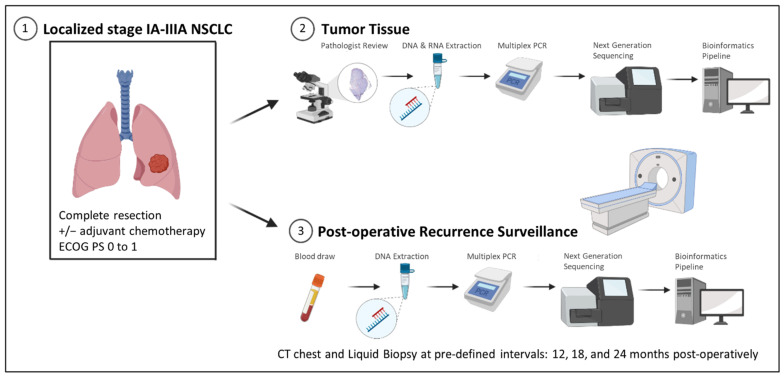
Study Design. Depicted is the real-world study design, whereby resectable non-small cell lung cancer patients underwent complete surgical resection. Formalin-fixed paraffin-embedded tumor tissue was subjected to targeted panel next-generation sequencing to detect genetic alterations. Routine post-operative CT chest surveillance was accompanied by targeted panel next-generation sequencing of plasma cell-free DNA to assess for the presence of circulating tumor DNA from a recurrence of the original tumor. ECOG: Eastern Cooperative Oncology Group.

This study was approved by the University of British Columbia Clinical Research Ethics Board (H18-03295). Molecular, demographic, and smoking status variables were prospectively collected for consecutive cases of early-stage (I–IIIA per American Joint Committee on Cancer 8th Edition staging [9]) NSCLC that were resected between May 2019 and February 2022 (Figure 1). All included tumors were ≥10 mm in maximal diameter and at least partially solid on the pre-operative CT chest. The included patients had Eastern Cooperative Oncology Group (ECOG) scores of 0 or 1. Post-operatively, patients with stage IB and higher disease on final surgical pathology were assessed by a medical oncologist for adjuvant systemic therapy, per local standard of care. Surveillance imaging for up to 24 months following surgery was reviewed. Chest CT or positron emission tomography (PET) imaging within 1 month of the 2-year anniversary of the date of the surgery was considered a ’24-month’ follow-up. Smoking history was assessed through a questionnaire completed by participants or through chart review.

### 2.2. Blood Processing and ctDNA Extraction

Blood samples (30 mL) were collected in Streck^TM^ blood collection tubes by intravenous puncture pre-operatively and at 12, 18, and 24 months after surgery, when possible. Each blood tube was centrifuged for 15 min at 1600× *g* to separate blood into plasma, buffy coat, and red cell components within 1–3 days of sample collection. The plasma was centrifuged a second time and stored at −80 °C until ready for extraction. To obtain total cell-free DNA (cfDNA), 6–12 mL of plasma from each patient was used with an optimized version of the Maxwell^®^ RSC Circulating DNA Purification Kit (Promega, Madison, WI, USA). The final cell-free DNA was quantified using the Qubit^®^ HS DNA kit (ThermoFisher, Waltham, MA, USA).

### 2.3. Tumor Tissue Specimen Collection and Extraction

The surgical specimens were processed into formalin-fixed paraffin-embedded (FFPE) blocks per standard clinical pathology practice. The tissue was sectioned from a representative FFPE tumor block, and DNA and RNA extractions were performed by Imagia Canexia Health (ICH, Vancouver, BC, Canada) using the GeneRead DNA FFPE kit (Qiagen, Hilden, Germany) and the Maxwell^®^ RSC RNA FFPE kit (Promega), respectively. Yields were quantified using Qubit^®^ DNA BR and Qubit^®^ RNA BR kits (ThermoFisher).

### 2.4. Targeted NGS Panels for NSCLC Genetic Alterations

Next-generation sequencing (NGS) of FFPE specimens was performed using the Find It^TM^ and Fusions^TM^ multiplex assays (ICH). Plasma cfDNA sequencing was performed using the Follow It^TM^ multiplex assay (ICH). The FFPE DNA-based Find It^TM^ and plasma cfDNA Follow It^TM^ assays used amplicon-based targeted multiplex NGS for the detection of single nucleotide variants (SNVs) and insertions and deletions (indels; up to 24 base pairs). The Find It^TM^ and Follow It^TM^ assays contained the same assay workflow and gene coverage (up to 39 genes covered depending on the assay version, listed in Supplementary Table S1). The Fusions^TM^ assay is an RNA-based gene partner agnostic multiplex NGS panel that can identify clinically relevant gene fusions (Supplementary Table S2).

In brief, the FFPE DNA or cfDNA was amplified in 2–3 separate primer pools using a maximum of 25 ng FFPE DNA or 5–33 ng of cfDNA in each primer pool. PCR template products were then pooled, purified, and amplified with unique dual indexes for sequencing. The final library pools consisted of 3 controls (Blank, Normal Female DNA, Horizon QMRS DNA positive control) and 21 test samples, which were sequenced on MiSeq or NextSeq500 instruments (Illumina, SanDiego, CA, USA) using the 300 cycle kit (2 × 150 bp). The Illumina MiSeq or NextSeq instrument was used for onboard demultiplexing for all data. Analysis pipelines used BWA to align to the human reference genome, GRCh37/hg19. Subsequent data filtering used proprietary artificial intelligence models trained to identify and filter true SNVs from artifacts and noise. Post-alignment indels were analyzed using Strelka [10]. Thresholds for SNV and indel reporting are shown in Supplementary Table S3. The Fusions^TM^ assay used cDNA reverse-transcribed from RNA and subjected to amplification, ligation, and PCR for targeted amplification. The libraries were amplified using unique dual index adapters (Illumina) and purified and sequenced using the MiSeq v2 300 cycle kits (Illumina). The in-house-developed fusion analysis pipeline identified unique fusion reads. Low-confidence fusions and exon-skipping events were excluded from reporting, except in cases with corresponding splice site SNVs identified in the DNA assay. An oncoprint including functional significance calls was generated using cBioPortal [11,12,13], excluding synonymous variants and classifying frameshifts as truncating mutations.

Variants identified in the Follow It^TM^ assay were filtered using the thresholds in Supplementary Table S3, which include a minimum SNV variant allele frequency (VAF) of 0.5% (version 4) or 0.3% (version 5) and a minimum indel VAF of 2% (version 4) or 0.7% (version 5). Gene coverage differences in versions 4 and 5 are summarized in Supplementary Table S1. Version 4 assays were used on 26 patients, version 5 assays were used on 33 patients, and a combination of version 4 and version 5 assays were used on 19 patients. Variants detected at ≥45% VAF in the buffy coat were excluded as not reflective of tumor DNA. Find It^TM^ and Follow It^TM^ assay results on the same patients were compared, and any matching variants were manually reviewed and verified. For the purposes of this study, only plasma samples with a ctDNA variant that matched a variant identified in the Find It^TM^ assay for the corresponding primary FFPE tumor were interpreted as ctDNA positive.

### 2.5. Statistical Methods

Categorical variables were compared using Fisher’s exact tests, and confidence intervals were calculated using the Wilson Score method. Recurrence-free survival was estimated using a survival curve and censored data. The clinical interpretation of imaging and biopsy results was used to define a recurrence, and the date of the first imaging evidence of recurrence was used as the date of recurrence. *p*-values < 0.05 were considered statistically significant. Statistical analyses were performed using R version 4.2.1 [14].

## 3. Results

### 3.1. Tumor Genetic Alterations and Pre-Operative ctDNA Assessment

**Figure 2:**
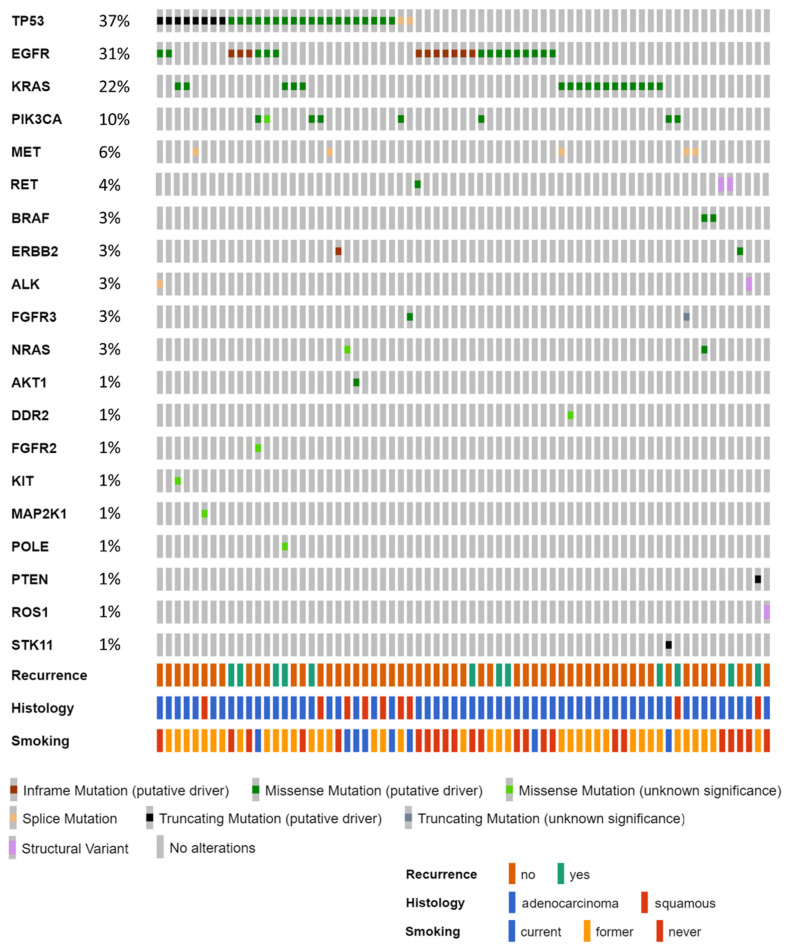
Oncoprint of alterations detected in DNA- and RNA-based assays on tumor resection specimens. The y-axis reports the alteration frequency of each gene out of all 78 patients. The nine patients with no identified alterations are not shown.

The surgical cohort included 78 patients (demographics in Table 1). All patients underwent complete surgical resection (R0). The panel-based DNA and RNA assays performed on FFPE tumor tissue from the resection specimen allowed the identification of at least one alteration in 69 out of 78 (88%) tumors (Figure 2). A variant was detected on the DNA assay for 65 patients, whereas four patients had an alteration detected only on the RNA assay (two RET fusions, one ALK fusion, and one ROS1 fusion). The most frequently mutated oncogenes in the study cohort were *EGFR* (31%) and *KRAS* (22%). Never-smokers were significantly enriched in *EGFR* mutations and *ALK*, *ROS1*, or *RET* fusions, which were present in 18 (62%) of the 29 never-smoking patients and only 10 (20%) of the 49 ever-smoking patients (difference = 42%, 95% CI: 17% to 61%, *p* = 0.0005). The incidence of DNA assay alterations (i.e., 65 out of 78 patients; 83%) is of interest, as tumor-informed ctDNA detection can only be performed on patients whose original tumor has known alterations that would also be detectable in the plasma if present.

A pre-operative liquid biopsy was obtained from 56 (86%) of the 65 patients for which mutations were identified in the tumor DNA assay. Liquid biopsies could not be obtained for the remaining nine patients due to COVID-19 restrictions or withdrawal of consent. In only one of these 56 cases was the same mutation detected in the plasma as in the tumor resection (a *TP53* R337L mutation, VAF 1.1% in the plasma), indicative of detectable circulating tumor DNA pre-operatively. The patient with pre-operatively detectable ctDNA had squamous cell carcinoma and did not experience recurrence within 24 months of follow-up. A liquid biopsy at 12 and 18 months after resection was negative for ctDNA in this patient.

### 3.2. Early Recurrence Following Surgical Resection

The incidence of recurrence during follow-up was assessed for the 65 patients with alterations in the tumor DNA assay, for whom a tumor-informed approach to ctDNA detection could be used. A total of 54 of these 65 patients had available CT imaging data at regular intervals up to and including the 24-month mark post-surgery. Of the remaining eleven patients, three died within 24 months of surgery (two deaths due to NSCLC, at 8 and 20 months after surgery), and eight patients were lost to imaging follow-up before 24 months; for these eight patients, the most recent CT or PET imaging was a median of 20 months after surgery (range 5–22 months). Fifteen patients had adjuvant chemotherapy following surgery when there was no evidence of recurrence.

Twenty-four-month recurrence-free survival was estimated at 82% (95% CI: 73–92%). Eleven (17%) of the 65 patients had imaging findings of recurrence, and six had biopsy-proven recurrence. The remaining five patients did not undergo biopsy as the imaging findings were sufficiently concerning to initiate treatment: one had brain lesions, one had regional lymph node enlargement with PET positivity, and three had lung lesions separate from the the original tumor resection site. The presenting pattern of recurrence was distant in five patients (54%; brain, leptomeningeal, bone, or adrenal) and local-regional in the remaining six patients (41%; pulmonary, pleural, and intrathoracic lymphadenopathy).

### 3.3. Post-Operative ctDNA Assessment

**Figure 3:**
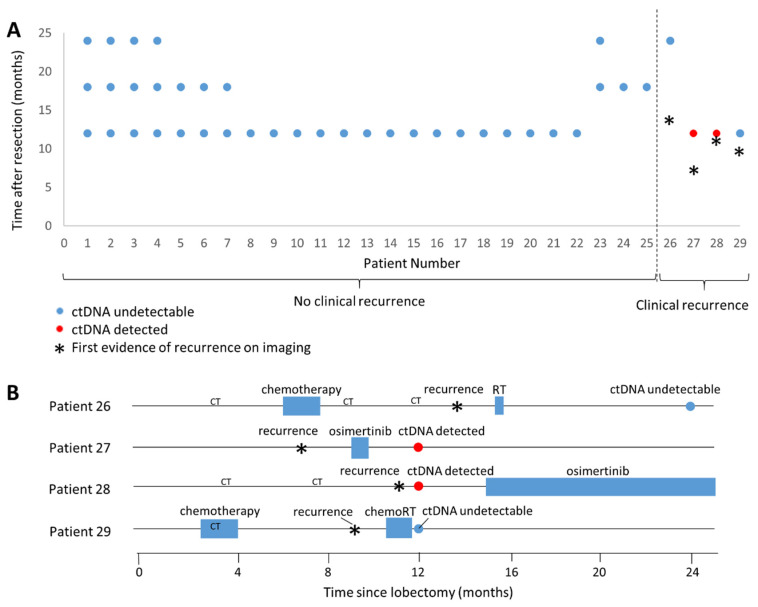
Timeline of cases with liquid biopsy. (**A**) Timepoints of liquid biopsy and recurrence in patients with post-operative liquid biopsy. All patients had 24 months of imaging follow-up after resection, except for patient 18, whose latest CT scan was 19 months after resection. ctDNA was collected at the indicated timepoints. (**B**) Treatments received by patients with recurrence relative to the timing of recurrence and liquid biopsy (circles). RT: radiation therapy; chemoRT: chemotherapy and radiation. ‘Recurrence’ refers to the date of the first imaging showing recurrence. ‘CT’ indicates the date of a chest CT scan with no evidence of recurrence. Only CT scans prior to recurrence are indicated.

Follow-up blood samples were collected from 29 of the 65 patients with alterations on the tumor DNA assay, with blood samples collected at 12, 18, and/or 24 months following surgical resection. Samples could not be collected from the remaining patients due to COVID-19 restrictions or withdrawal of consent. Twenty-one patients were only available to provide a blood sample at one of the above timepoints, most commonly at 12-month follow-up (Figure 3A).

Of the 29 patients with post-operative liquid biopsy, 4 (all adenocarcinomas) had imaging evidence of recurrence. Two of those four cases had evidence of ctDNA in the 12-month plasma sample, one with an *EGFR* p. (L747_S752del) in both the tumor and plasma (plasma VAF 2.65%) and the other with an *EGFR* p. (E746_A750del) in both the tumor and plasma (plasma VAF 1.1%). The first imaging evidence of recurrence was detected at 7 months after surgery in the former patient (ipsilateral pleural nodules measuring up to 3 cm with loculated pleural effusion and possible involved mediastinal and superior abdominal lymph nodes) and 11 months after surgery in the latter patient (pleura, rib, ileum, vertebrae, and mediastinal metastases, with masses measuring up to 2.6 cm). Both had recurrence confirmed by tissue biopsy. One case received a three-week trial of osimertinib that was discontinued for adverse reactions two months before the plasma sample was collected, whereas the other did not have osimertinib treatment until three months after the plasma sample was collected (Figure 3B).

The two patients who were considered clinically to have recurrence but had no evidence of ctDNA on plasma sequencing both only had imaging (not biopsy) evidence of recurrence. In both cases, the patient received treatment prior to the collection of the blood sample for ctDNA testing, which may have affected the results. The liquid biopsy for one of these patients, who had an *EGFR* L858R mutation, was collected eight months after curative intent stereotactic radiation treatment of a 1.2 cm PET-positive lung nodule ipsilateral to the original tumor but in a different lobe (the original tumor was removed with lobectomy). The other patient had *PIK3CA* and *TP53* variants on tumor tissue sequencing and had PET-positive enlarged superior mediastinal lymph nodes measuring up to 1.5 cm treated with radiation and chemotherapy, with treatment completed prior to the liquid biopsy.

Of the remaining 25 patients with a post-operative liquid biopsy but no clinical recurrence within 24 months, none had variants detected in the plasma that were also present in the tumor tissue sequencing. Thus, there were no false positive indications of recurrence based on plasma sequencing in our cohort.

## 4. Discussion

Recurrence following curative-intent surgery for early-stage disease occurs in a considerable proportion of NSCLC patients [1]. Targeted systemic therapy may be indicated upon demonstration of recurrence for tumors driven by alterations involving *EGFR* (epidermal growth factor receptor), *ALK* (anaplastic lymphoma kinase), *MET* (Met proto-oncogene), *RET* (Ret proto-oncogene)*,* or *ROS1* (c-ROS proto-oncogene 1) [15,16,17,18,19,20,21,22]. Definitive confirmation of tumor recurrence and determination of whether targetable alterations are present in the recurrent tumor can, therefore, be critical for appropriate management.

Specialized methods have been developed for ultra-sensitive detection of ctDNA for minimal residual disease detection but typically require additional or proprietary methodological steps, including the addition of unique molecular barcodes for error correction, duplex sequencing, ultra-deep sequencing, patient-specific tumor-informed panels, and bioinformatics analysis and tools [23]. These methodologies can increase the complexity of clinical validation for these assays, which may only be available on a private-pay basis directly through companies. In contrast, targeted amplicon panel sequencing workflows already validated for FFPE tumor tissue can be adapted for use on plasma cell-free DNA, allowing for simplified in-house assay development, albeit typically with lower sensitivity than assays designed for minimal residual disease detection, which typically have a lower limit of detection of ~0.01% VAF or lower. The ICH Follow-It^TM^ assay used in this study is one example of an amplicon panel sequencing approach where similar laboratory methodologies were used for both plasma cfDNA and FFPE tissue DNA, although the bioinformatic analysis pipelines allowed for different thresholds of SNV detection between FFPE (1% VAF) and cfDNA (0.3% VAF). The ICH Follow-It^TM^ plasma ctDNA assay was developed primarily for metastatic cancers to allow for the identification of ctDNA mutations for targeted therapy, assessment of resistance mutations following suspected or known relapse, or tracking the response of metastatic disease to treatment. A limitation of this assay was that it was not designed for ultra-sensitive minimal residual disease detection in patients without radiologic evidence of disease, as assays for minimal residual disease typically have a lower limit of detection (~0.01% VAF or lower) that is lower than that of the ICH Follow-It^TM^ assay (0.3% VAF for SNVs) [23]. There is limited literature on whether this type of lower-sensitivity ctDNA assessment has real-world utility for confirmation of recurrence after suspicious findings are identified on imaging. There are also limited data on the utility of this type of assay employed pre-operatively to guide neo-adjuvant treatment considerations.

The present study demonstrates that using a targeted panel assay, ctDNA can be detected in a subset of patients who have recurrence of previously resected early-stage NSCLC. In two out of four cases with recurrence, plasma ctDNA mutations were identifiable at 1 month and 5 months, respectively, after the first evidence of recurrence was detected on imaging. These findings support the notion that a ctDNA assay not designed for minimal residual disease could potentially detect plasma ctDNA when radiologic evidence of recurrence is present and thus could potentially serve as a usual tool for confirmation of suspected recurrence.

There were no false positive indications of recurrence on liquid biopsy in the 25 recurrence-free cases, likely in part due to the tumor-informed approach: only variants in the plasma sample that were also present in the resected tumor were considered evidence of ctDNA. A limitation of this tumor-informed approach is that ctDNA would not be recognized as such in a patient whose tumor tissue had no detectable alterations in the limited panel used, corresponding to 17% of the tumors sequenced as part of this study. Notably, the proportion of tumors with identifiable mutations may be greater if a more comprehensive panel is used. Using a tumor-informed approach, ctDNA from a second primary malignancy would also not be recognized as such except in the unlikely event that it shares a variant with the prior tumor, in which case it would falsely be assumed to be a recurrence of the original malignancy. The odds of separate primary tumors having mutations in common are relatively low, such that it is typically reasonable to interpret ctDNA with a mutation matching the prior tumor as evidence of recurrence. As biopsy morphology has limited accuracy in distinguishing a recurrence from a separate primary adenocarcinoma, the capacity of ctDNA to provide evidence in support of recurrence is a potential advantage compared to performing biopsy alone.

Both cases with ctDNA detected following resection had imaging evidence of recurrent disease involving multiple sites, likely contributing to ctDNA levels being high enough for detection. Prior studies have found that ctDNA is less likely to be detected in cases with more localized or small-volume disease [24,25]. In keeping with this notion, the two patients with ‘false negative’ ctDNA results after possible recurrence had only limited evidence of disease on imaging and no biopsy confirmation of recurrence. These two patients also underwent treatment for the suspected recurrence prior to testing for ctDNA, and one had a long (8-month) interval between radiation completion and liquid biopsy, potentially further reducing ctDNA levels.

Increasing interest in neo-adjuvant treatment for early-stage NSCLC [26] has raised consideration for biomarker testing prior to tumor resection, potentially through analysis of ctDNA. However, pre-operative liquid biopsy in this study detected ctDNA in only 1 out of 56 cases with detectable tumor DNA alterations. In contrast, high-sensitivity ctDNA assays have detected ctDNA prior to resection in ~24–60% of early-stage NSCLC cases [25,27,28], underscoring the need for high-sensitivity assays in the pre-operative early-stage scenario. Of note, the one patient with pre-operatively detected ctDNA in this study had squamous cell carcinoma, and squamous cell carcinomas have been associated with greater shedding of ctDNA than adenocarcinomas [24,25].

The limitations of this study include the small sample size, limited follow-up time, limited number of ctDNA samples, and small number of recurrences. Additional studies of patients with small-volume recurrent disease and no intervening treatment prior to liquid biopsy are needed to determine sensitivity in this population. The timing of liquid biopsy relative to the appearance of recurrent disease on imaging was variable given our use of scheduled patient follow-ups and considerable loss to follow-up, in part related to COVID-19 restrictions. Generalizability to populations with different clinical and pathologic demographics, whose tumors may have different propensities to shed ctDNA, also remains to be established.

This proof-of-concept real-world prospective study demonstrates that tumor-informed amplicon panel sequencing applied to plasma cell-free DNA provides high-confidence molecular confirmation of NSCLC recurrence in a subset of cases, even when the assay’s lower limit of SNV detection is 0.3% VAF, higher than most assays designed for minimal residual disease detection (typically ~ 0.01% VAF or lower). This study is a preliminary step in demonstrating the clinical utility of a liquid biopsy workflow for the confirmation of recurrence in patients with previously resected early-stage NSCLC. Invasive tissue diagnosis may be avoidable in such patients, reducing complications and supporting the timely initiation of treatment. Further study is necessary to define the sensitivity of different ctDNA detection approaches in this clinical setting and to inform post-operative oncologic surveillance guidelines for lung cancer.

## Figures and Tables

**Table 1:** Description of Study Participants. Patients whose tumors had one or more detectable DNA alteration in the DNA assay were assessed for recurrence during the 24-month follow-up.

Demographics/Diagnosis	All *n* = 78	Alteration Detected in DNA Assay *n* = 65	No Recurrence *n* = 54	Recurrence *n* = 11
Age (years): median (range)	69.5 (43–83)	71 (43–83)	73 (48–83)	64 (43–79)
Female: *n* (%)	46 (59)	38 (58)	31 (57)	7 (64)
Smoking status: *n* (%)				
Never smoker	29 (37)	19 (29)	17 (31)	2 (18)
Former smoker	41 (53)	38 (58)	29 (54)	9 (82)
Current	8 (10)	8 (12)	8 (15)	0 (0)
Histology: *n* (%)				
Adenocarcinoma	68 (87)	56 (86)	47 (87)	9 (82)
Squamous cell carcinoma	10 (13)	9 (14)	7 (13)	2 (18)
Overall stage *n* (%)				
IA1	11 (14)	8 (12)	8 (15)	0 (0)
IA2	22 (28)	18 (28)	18 (33)	0 (0)
IA3	9 (12)	5 (8)	5 (9)	0 (0)
IB	12 (15)	12 (18)	9 (17)	3 (27)
IIA	0 (0)	0 (0)	0 (0)	0 (0)
IIB	14 (18)	13 (20)	9 (17)	4 (36)
IIIA	10 (13)	9 (14)	5 (9)	4 (36)
Extent of surgical resection				
Wedge resection	15 (19)	11 (17)	10 (19)	1 (9)
Segmentectomy	1 (1)	0 (0)	0 (0)	0 (0)
Lobectomy	58 (74)	50 (77)	42 (78)	8 (73)
Bi-lobectomy	3 (4)	3 (5)	2 (4)	1 (9)
Pneumonectomy	1 (1)	1 (2)	0 (0)	1 (9)

## Data Availability

The original contributions presented in the study are included in the article/Supplementary Material; further inquiries can be directed to the corresponding author.

## Supplementary Materials

The following supporting information can be downloaded at: https://www.mdpi.com/article/10.3390/curroncol31070302/s1, Supplementary Table S1: FIND ITTM and FOLLOW ITTM Panels; Supplementary Table S2: FUSIONSTM Panel Version 1.0; Supplementary Table S3: Thresholds for reporting variants.
