# Supplementary material for: Confirmation of Recurrent Lung Cancer Following Resection Using Liquid Biopsy, a Proof-of-Concept Real-World Study
Source: Curr Oncol. 2024 Jul 17;31(7):4052–62. doi: 10.3390/curroncol31070302 (PMC11276127; doi:10.3390/curroncol31070302)
Supplement: Supplementary file 1 [file curroncol-31-00302-s001.zip › curroncol-3084737-supplementary.pdf]

**Supplementary Table 1:** FIND IT$^{TM}$ and FOLLOW IT$^{TM}$ Panels

| Gene | Positions covered in version 4 | Additional positions covered in version 5 |
|---|---|---|
| AKT1 | E17 | No changes |
| ALK | T1151, L1152, C1156, F1174, L1196, L1198, G1202, D1203, S1206, G1269, R1275 | Y1278 |
| AR | L702H, S741, W742, V716, H875, F877, T878 | M896 |
| BRAF | Q201, G466, F468, G469, Y472, D594, G596, L597, V600, K601 | part of Exon15 (V600-M620), G606 |
| CCNE | none | No changes |
| CTNNB1 | D32, S33, G34, S37, T41, S45 | No changes |
| DDR2 | L239, I638, S768 | No changes |
| DICER | none | D1705-D1709, G1809, D1810-E1813 |
| EGFR | S492, Exon18, Exon19, Exon20, Exon21 | R108, A289, P596, G598, |
| ERBB2 | G309, S310, C805, Exon20 | K753, L755, I767, D769, |
| ESR1 | K303, S463, V534, P535, L536, Y537, D538 | E380 |
| FGFR1 | Not covered | N546, K656 |
| FGFR2 | Not covered | S252, P253, W290, A315, S372, Y375, C382, N549, K659, E731, E777 |
| FGFR3 | Not covered | R248, S249, G370, S371, Y373, G380, A391, K650 |
| FOXL2 | Not covered | C134 |
| GNA11 | Q209 | No changes |
| GNAQ | Q209 | No changes |
| GNAS | R201 | No changes |
| HRAS | G12, G13, Q61 | No changes |
| IDH1 | R132 | No changes |
| IDH2 | R140, R172 | No changes |
| KIT | Exon9, Exon11, Exon13, T670, D816, D820, N822, Y823, A829 | S476, Y553, W557,, V559, V560, L576, K642, V654 |
| KRAS | G12, G13, A59, Q61, K117, A146 | K5, A11, L19, Q22, G60 |
| MAP2K1(MEK1) | Q56, K57, K59, D67, C121, P124, P387 | F53, V60, I103, I111, N122, |
| MAP2K2(MEK2) | F57, Q60, K61, L119 | H123, G132 |
| MET | Y1253, Exon13, Exon 14 (-50 to +25), Exon18 | T1010, V1112, H1112, G1181, L1213, D1246, Y1248 |

| | | |
|---|---|---|
| NRAS | G12, G13, A59, Q61, K117, A146 | G60 |
| NTRK1 | Not covered | F589, G595, G667 |
| NTRK3 | Not covered | G623, G696 |
| PDGFRA | N659, R560-E571, D842, L839-Y849 | P577 |
| PIK3CA | R88, E542, E545, Q546, D549, M1043, N1044, A1046, H1047, G1049 | C90, R93, P104, G106, N107, R108, K111, R115, N345, R357, G364, E365, Exon6 [start to P377], C420, E453, P539, E970, E978 |
| POLE | Exon9, Exon10, Exon11, Exon12, Exon13, Exon14 | No changes |
| PTCH1 | W844, G1093 | No changes |
| PTEN | R130 | A126, G129, R173, R233, K254-K267 |
| RET | C634, V804, M918 | G533, K603, C609, C611, C618, C620, C630, D631, G691, E768, L790, Y791, Y806, A883, R886, S904, A919, Exon10, Exon 13, Exon15 |
| ROS1 | L2026, G2032 | S1986 |
| STK11 | Not covered | Q37, P281 |
| SMO | D473, S533, W535 | removed |
| TP53 | Exon4, Exon5, Exon6, Exon7, Exon8, Exon9 | No changes |

**Supplementary Table 2:** FUSIONS$^{TM}$ Panel Version 1.0

| **Gene** | **Targeted Exons** |
|---|---|
| ALK | 2, 10, 16, 18, 19, 20 |
| BRAF | 8, 9, 10, 11, 15 |
| CSF1 | 6 |
| EGFR | 1, 20, 21, 24, 27 |
| FGFR1 | 1, 2 |
| FGFR2 | 10, 17 |
| FGFR3 | 4, 17, 18 |
| MET | 2, 15 |
| NRG1 | 2, 6 |
| NTRK1 | 2, 7, 8,10-12 |
| NTRK2 | 10-15 |
| NTRK3 | 7, 14, 15, 18 |
| PDGFB | 2 |
| PPARG | 2 |
| RAF1 | 8 |
| RET | 2, 7-12 |
| ROS1 | 11, 32-36 |

**Supplementary Table 3:** Thresholds for reporting variants. The threshold for all three parameters for each variant were required to be met.

| Variant type | Parameter | Version 4 | | Version 5 | |
|---|---|---|---|---|---|
| | | Plasma | FFPE | Plasma | FFPE |
| SNV | Probability score | ≥ 0.7 | ≥ 0.7 | ≥ 0.7 | ≥ 0.7 |
| | VAF | ≥ 0.5 | ≥ 0.7 | ≥ 0.3 | ≥ 0.7 |
| | Coverage | ≥ 500 | ≥ 500 | ≥ 150 | ≥ 150 |
| indel | Probability score | ≥ 400 | ≥ 400 | ≤ 0.39 | ≤ 0.39 |
| | VAF | ≥ 2 | ≥ 2 | ≥ 0.7 | ≥ 0.7 |
| | Coverage | ≥ 500 | ≥ 500 | ≥ 5000 | ≥ 5000 |
